# Efficacy and Safety of Oral Neomycin for the Decolonization of Carbapenem-Resistant *Enterobacterales*: An Open-Label Randomized Controlled Trial

**DOI:** 10.3390/antibiotics13080781

**Published:** 2024-08-20

**Authors:** Lalita Tancharoen, Ananya Srisomnuek, Surapee Tiengrim, Narisara Thamthaweechok, Teerawit Tangkorskul, Visanu Thamlikitkul

**Affiliations:** 1 Department of Medicine, Faculty of Medicine Siriraj Hospital, Mahidol University, Bangkok 10700, Thailand; lalita33ae@gmail.com (L.T.); surapee.tie@mahidol.ac.th (S.T.); narisara.tha@mahidol.ac.th (N.T.); teerawit.tan@mahidol.ac.th (T.T.); 2 Department of Research, Faculty of Medicine Siriraj Hospital, Mahidol University, Bangkok 10700, Thailand; ananya.sri@mahidol.ac.th

**Keywords:** carbapenem-resistant *Enterobacterales*, colonization, CRE, decolonization, neomycin

## Abstract

Background: Patients with carbapenem-resistant *Enterobacterales* (CRE) in the gastrointestinal (GI) tract are at risk for subsequent infections and transmission, necessitating contact precautions. Neomycin has shown in vitro activity against CRE in 66–85% of isolates. This study evaluated the efficacy and safety of neomycin for CRE decolonization. Methods: In this open-label randomized controlled trial, stool/rectal swab samples from high-risk patients were collected and tested for CRE colonization in the GI tract. Patients who had CRE and met eligible criteria were divided into a neomycin group (*n* = 26; treated with 4.2 g/day neomycin for 5 days) and a control group (*n* = 26). CRE detection in stool/rectal swabs was performed on days 7 ± 2 and 14 ± 2. Results: The two groups’ baseline characteristics were similar. CRE presence on day 7 ± 2 was significantly lower in the neomycin group (46.2%) than in the control group (80.8%, *p* = 0.01). Efficacy of neomycin (4.2 g/day for 5 days) for CRE decolonization was 42.8–53.8% by day 7. By day 14 ± 2, the CRE rate in the neomycin group had risen to align with the control group’s rate (73.1% vs. 61.5%, *p* = 0.56). The neomycin group experienced mild, temporary, gastrointestinal side-effects. Conclusions: Neomycin effectively reduced CRE colonization on day 7 ± 2, but its impact waned by day 14 ± 2. This suggests that neomycin dosage was too low and the duration of treatment was too short for lasting CRE decolonization.

## 1. Introduction

The identification of carbapenem-resistant *Enterobacterales* (CRE), a critical priority pathogen, requires intensified research efforts to develop effective countermeasures [1]. Carbapenem resistance primarily arises from carbapenemase production within *Enterobacterales*. CRE frequently colonizes the gastrointestinal (GI) tract in asymptomatic carriers and infected individuals. Efforts to mitigate CRE emergence and transmission include robust antibiotic stewardship and infection prevention and control strategies [2]. Nevertheless, the global and local prevalence of CRE infections in healthcare settings continues to rise [3,4]. Therapeutic options for CRE infection are limited to certain older agents, like polymyxins, and newer antibiotics, such as cefiderocol and ceftazidime/avibactam, which are effective against CRE [5,6,7,8]. However, cefiderocol is unavailable in Thailand. Additionally, ceftazidime/avibactam is expensive and ineffective against CRE strains harboring the New Delhi metallo-beta-lactamase (NDM) gene [9].

Mortality from CRE infections exceeds that of infections caused by carbapenem-susceptible *Enterobacterales*, with Siriraj Hospital reporting rates between 47.7% and 68.3% from 2015 to 2020 [6,7,8,9,10,11]. Fecal CRE carriage risks include potential subsequent CRE infections, transmission of CRE to others in hospital settings, and the necessity for extensive contact precautions [11,12,13,14]. Therefore, GI tract CRE decolonization is critical. Studies on CRE decolonization [15,16,17,18,19,20,21] have mainly involved oral antibiotics such as colistin and gentamicin, which have shown an efficacy ranging from 33% to 83% [16,17,18]. However, resistance to these antibiotics in patients’ GI flora has been documented [17,19,20].

Colistin and gentamicin, while typically administered parenterally for Gram-negative infections, lack formal endorsement for oral use in CRE decolonization [15,16,17,18,19,20,21,22,23]. Fecal microbiota transplantation has shown CRE decolonization efficacy rates of 61% at 1 month and 78.7% at 6–12 months across various studies, including a small randomized controlled trial (RCT) [24]. However, the complex procedures required for fecal microbiota transplantation limit its practicality, especially in Thailand.

Neomycin, an oral aminoglycoside with minimal GI absorption (≤3%), largely remains in the GI tract and exits unchanged in stool. This antibiotic is recommended for infection prophylaxis in colorectal surgery, paired with erythromycin or metronidazole, and for treating hepatic coma at 4–12 g daily for 5–6 days [25]. In China, 65.7% of 134 CRE isolates were neomycin-susceptible (minimum inhibitory concentration, MIC ≤ 8 mg/L), while the susceptibility rates to amikacin (55.2%) and gentamicin (28.4%) were lower. However, many of these isolates were *Klebsiella pneumoniae* carbapenemase (KPC)-producing, which is rare in Thailand [26,27]. A Thai study showed that 85.2% of 109 CRE strains were neomycin-susceptible, with isolates primarily containing oxacillinase (OXA) and NDM enzymes but not KPC [28].

An RCT evaluated a regimen combining oral neomycin (1.4 g) and colistin sulfate (8 million IU) for 5 days with fecal microbiota transplantation. This regimen was somewhat effective for CRE decolonization, showing a decolonization rate of 41% compared to 29% in the control group. However, the difference in clinical outcomes was not statistically significant, with an odds ratio (OR) of 1.7 and a 95% confidence interval (CI) of 0.4–6.4 [21]. Another RCT focusing on CRE decolonization in solid organ transplant recipients using neomycin (1 g) and colistin sulfate (5.04 million IU) for 14 days showed no significant difference in multidrug-resistant bacterial infection rates within 30 days compared to a non-antibiotic group (9.4% vs. 13.5%, *p* = 0.517) [19]. Currently, no studies have explored the use of oral neomycin alone for CRE decolonization in individuals with fecal CRE carriage.

This study evaluated the efficacy and safety of neomycin for short-term CRE decolonization in patients with fecal CRE carriage. Secondary objectives included investigating carbapenem resistance mechanisms in CRE isolates and assessing the susceptibility of CRE isolates from participants’ stool or rectal swab samples to neomycin, gentamicin, and amikacin.

## 2. Results

### 2.1. Inclusion of Participants

**Figure 1:**
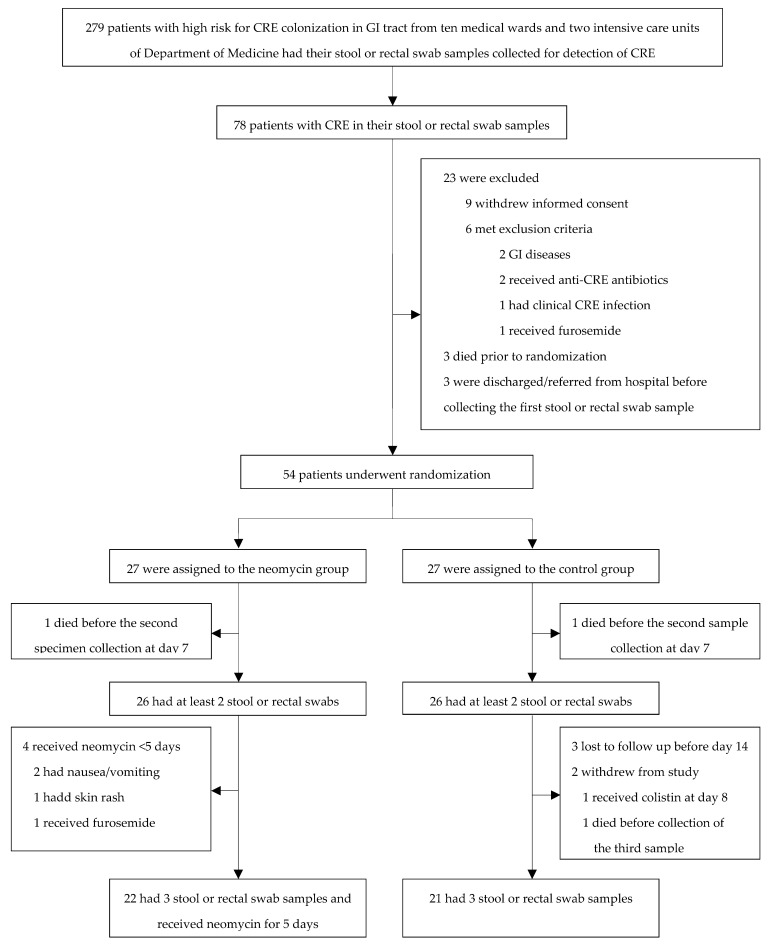
Participant screening and randomization flowchart for the modified intention-to-treat cohort.

Figure 1 illustrates the inclusion flowchart for the modified intention-to-treat (mITT) population and per-protocol (PP) population. Of 279 high-risk hospitalized patients screened, 78 (28.0%) tested positive for CRE. The mITT population comprised 52 patients who met the eligibility criteria and was evenly split between the neomycin (*n* = 26) and control (*n* = 26) groups. The PP population comprised 43 patients who had three stool or rectal swab samples and received neomycin for 5 days (22 patients in the neomycin group) and those who had three stool or rectal swab samples (21 patients in the control group).

### 2.2. Baseline Characteristics of Participants 

There were no significant differences in baseline characteristics among the 52 participants in the mITT population (Table 1). The average age was 63.6 years in the neomycin group and 71.7 years in the control group (*p* = 0.11). Male participants constituted 30.8% of the neomycin group versus 57.7% of the control group (*p* = 0.05). Most participants had at least one underlying condition (84.6% neomycin vs. 100% control, *p* = 0.11). Both groups had a median Karnofsky score of 50. Approximately 80.8% of patients in the neomycin group had documented or presumed infections at the time of admission, while 100% of patients in the control group had documented or presumed infections (*p* = 0.05).

Throughout their current admission until the end of the study, all participants in both groups were on antibiotics that were ineffective against CRE. The most frequently administered antibiotics were piperacillin–tazobactam (63.5%), meropenem (51.9%), ceftriaxone (42.3%), and levofloxacin (23.1%). Common interventions included indwelling urinary catheters and mechanical ventilation. At baseline, 28 CRE isolates were identified in the stool or rectal swabs of each group. In the neomycin group, 8 were *E. coli* (28.6%) and 20 were non-*E. coli* (71.4%); in the control group, 10 were *E. coli* (35.7%) and 18 were non-*E. coli* (64.3%), with no significant difference between groups (*p* = 0.78). In both groups, two participants had both *E. coli* and non-*E. coli* CRE isolates in a single sample. The non-*E. coli* isolates were predominantly *K. pneumoniae* or *Klebsiella* spp., with 14 isolates in the neomycin group and 16 in the control group (*p* = 0.78).

In the neomycin group, the susceptibility rates of the CRE isolates to neomycin, gentamicin, and amikacin were 92.9%, 50.0%, and 46.4%, respectively, similar to those in the control group (92.9%, 46.4%, and 53.6%, respectively). In both groups, the MIC_50_ and MIC_90_ for neomycin against CRE isolates were 2 mg/L and 8 mg/L, respectively. Among the 45 carbapenemase-resistant CRE isolates detected on day 1, OXA-48 alone (37.8%), OXA-48 with NDM (20.0%), and NDM alone (20.0%) were the most prevalent, with no KPC found. The activity of neomycin against CRE isolates with OXA-48 and/or NDM was comparable to that against CRE isolates without these carbapenemases (97.1% vs. 90.5%, *p* = 0.55).

### 2.3. Efficacy of Neomycin for the Decolonization of CRE at Day 7 ± 2 and Day 14 ± 2

Table 2 details the efficacy of neomycin for CRE decolonization. On day 7 ± 2, 46.2% of participants in the neomycin group had CRE, compared to 80.8% in the control group (*p* = 0.01), indicating a 53.8% decolonization efficacy for neomycin from baseline and 42.8% relative to the control. By day 14 ± 2, CRE detection in the neomycin group rose to 73.1%, with all 26 participants providing samples, while five samples from the control group were missing.

Assuming that the five missing samples in the control group were CRE-negative, the detection rate would adjust to 61.5%, closely matching the 73.1% in the neomycin group and without a significant difference (*p* = 0.56). Conversely, presuming that these samples were CRE-positive, the control’s detection rate would align with the initial 80.8%, still not significantly different from the neomycin group’s 73.1% (*p* = 0.74). Nearly all of the CRE species identified on days 7 ± 2 and 14 ± 2 matched those found on day 1, with only two participants exhibiting different CRE species by day 14 ± 2.

### 2.4. Species of CRE Isolates

Of the 130 CRE isolates recovered from stool or rectal swab samples on days 1, 7 ± 2, and 14 ± 2, 36 were *E. coli* isolates (27.7%) and 94 were non-*E. coli* isolates (72.3%). Four subjects presented with two CRE species in a single sample. The non-*E. coli* isolates primarily consisted of *K. pneumoniae* or *Klebsiella* spp. Almost all of the CRE species identified on days 7 ± 2 and 14 ± 2 matched those from day 1. However, one participant in the neomycin group and one in the control group exhibited different CRE species on day 14 ± 2 compared to day 1.

### 2.5. Safety and Tolerability

Among the 26 participants in the neomycin group, half experienced adverse events, predominantly mild and self-resolving, including diarrhea (10 patients), nausea (4), nausea with vomiting (2), and one instance of skin rash, with no reported ototoxicity or nephrotoxicity. In contrast, the control group reported no adverse events. Two participants in the neomycin group stopped treatment due to nausea or vomiting on the first day, and neomycin was discontinued for another participant due to a skin rash, which was deemed unrelated to the medication by the attending physician and clinical course of the rash. Despite experiencing diarrhea, 10 participants in the neomycin group had mild symptoms that required no treatment, and all chose to continue in the study.

### 2.6. Susceptibility Rates of CRE Isolates

Table 3 presents the susceptibility rates of the CRE isolates to neomycin, gentamicin, and amikacin on days 1, 7 ± 2, and 14 ± 2. At these time points, there were no significant differences in the susceptibility rates to any of the three antibiotics. Likewise, no significant differences were observed in the susceptibility rates between the neomycin and control groups on any of the collection days. At day 7 ± 2, the MIC_50_ and MIC_90_ values for neomycin were 8 mg/L and 16 mg/L, respectively, in the neomycin group. In the control group, the values were 2 mg/L and 8 mg/L, respectively. By day 14 ± 2, the MIC_50_ and MIC_90_ for neomycin were 4 and 32 mg/L, respectively, in the neomycin group, but the MIC_50_ and MIC_90_ values remained at 2 and 8 mg/L, respectively, in the control group.

## 3. Discussion

This study aimed to decolonize CRE in patients’ GI tracts to prevent further infections and transmission to others and reduce the costs associated with contact precautions with neomycin alone. CRE detection was conducted using stool or rectal swab samples as representatives of intestinal content, with a preference for stool samples. Rectal swabs were used only when stool samples were unavailable or if a participant was discharged on the scheduled collection day without providing a stool sample.

Although detecting CRE in a stool or rectal swab sample confirmed a patient’s CRE carriage, a negative result did not definitively indicate the absence of CRE, potentially due to sampling errors. Given this study’s RCT design and consistent sample collection protocol, such errors were expected to be evenly distributed across both groups, mitigating bias. Initial enrollment was based on rapid test results from carbapenem-resistant selective agar, which was deemed reliable based on our experience. CRE species identification and carbapenem resistance verification were subsequently conducted using ertapenem disk testing. The study targeted OXA-48 and NDM carbapenemases (prevalent in Thailand [27]) and KPC (based on a Chinese study indicating the differential activity of neomycin against various CRE carbapenemases [26]).

Neomycin was chosen for CRE decolonization over oral colistin or gentamicin due to its minimal absorption, ensuring that it remains and is excreted unchanged in the GI tract. Its higher activity against CRE compared to gentamicin and amikacin suggests a distinct resistance mechanism [26,28,29]. The Clinical and Laboratory Standards Institute lacks specific neomycin susceptibility cutoffs for CRE, so the French Society of Microbiology guidelines were used. Oral colistin and gentamicin have been linked to resistance development in intestinal Gram-negative bacteria [17,19,20], and their parenteral forms are ineffective against such resistant strains, unlike neomycin, which lacks a parenteral option for systemic infections. The neomycin dosage and treatment duration in this study were aligned with the lowest dose and shortest duration recommended for hepatic coma, a regimen long utilized without notable serious adverse events [25]. This conservative approach was adopted to address safety concerns associated with the novel application of neomycin monotherapy at a higher dosage of 4.2 g. Such a dosage surpasses those utilized in combination therapies in earlier studies [19,21]. Approximately 75% of the CRE isolates in this study had OXA-48 and NDM, with the remaining isolates potentially resistant to alternate carbapenemases or possessing different resistance mechanisms [30].

On day 7 ± 2, the CRE colonization rate in the neomycin group was significantly lower than that in the control group, indicating a decolonization efficacy ranging from 42.8% to 53.8%. This effect is attributed to neomycin, as patients receiving CRE-active antibiotics were not included in the study. The 46.2% CRE persistence rate in the neomycin group may be related to a suboptimal neomycin dose, given that 92.9% of the baseline CRE isolates were neomycin-susceptible, yet many persistent isolates showed high MICs of neomycin. Uneven distribution of neomycin in the intestinal tract could have resulted in varied neomycin concentrations across different sections, potentially leaving some areas untreated and allowing for the persistence of CRE isolates with low MICs of neomycin. The changing volume of intestinal content suggests that dosing that is more frequent than three times daily might maintain consistent neomycin levels. Thus, increasing both the dose and frequency of neomycin administration could enhance the concentration and distribution of neomycin in the intestinal contents, potentially improving decolonization efficacy.

On day 14 ± 2, the CRE colonization rates in both groups exceeded 60%, an increase from day 7 ± 2. This could stem from several factors, including the possibility that the 5-day neomycin treatment was insufficient to sustainably inhibit or eliminate the CRE isolates, which by day 14 ± 2 were typically the same species identified on days 1 and 7 ± 2. Thus, a longer neomycin treatment duration may be necessary. The lower CRE persistence rate in the control group (61.5%) than in the neomycin group (73.1%) on day 14 ± 2 may not hold statistical significance. This is because samples from five participants in the control group were missing, in contrast to the neomycin group, from which samples were obtained from all participants.

Although decolonization of CRE from the stool of individuals with CRE colonizers may be transient, the period of an absence of CRE after CRE decolonization is still useful for some particular patients. The risk of developing CRE infection and mortality of the patients with hematologic malignancy without CRE in their stool after CRE decolonization who received chemotherapy was decreased when compared with the patients with CRE colonizers [18]. 

In the worst-case scenario, if the missing samples from the five control group participants on day 14 ± 2 all contained CRE, the group’s CRE persistence rate would increase to 80.8%. However, all five missing samples being CRE-free should be improbable, given that CRE was detected in these participants on days 1 and 7 ± 2. The emergence of different CRE species by day 14 ± 2 in two participants might be attributed to antibiotic pressure, notably from meropenem, which was administered to 59.1% of participants. This could lead to carbapenem resistance in GI tract Gram-negative bacteria, underscoring the need for careful antimicrobial stewardship to prevent carbapenem overuse. Additionally, the new CRE species could originate from other patients, healthcare workers, or a CRE-contaminated environment in a hospital, highlighting the importance of strict adherence to infection prevention and control practices to prevent CRE acquisition.

In this study, the efficacy of neomycin in reducing CRE on day 7 ± 2 (42.8% to 53.8%) was similar to that reported in previous RCTs using oral colistin and/or gentamicin for 7–14 days [16,18]. However, these earlier studies noted a decline in efficacy 4–6 weeks after decolonization, with no significant difference between intervention and control groups. Another RCT focused on preventing multidrug-resistant *Enterobacterales* in solid organ transplant recipients by using oral colistin (50 mg, four times daily) and neomycin (250 mg, four times daily). The RCT revealed no significant difference in the risk of infection with multidrug-resistant *Enterobacterales* between the decolonization group (9.4%) and the non-decolonization group (13.5%, *p* = 0.52) [19]. A semi-RCT exploring three regimens (oral gentamicin, colistin, or both) over a median of 31 days of treatment in CRE-colonized patients showed significant differences in CRE eradication between the intervention and control groups (*p* < 0.01) but no significant variation in efficacy among the regimens [17].

The present study revealed that neomycin was significantly more active against CRE than gentamicin and amikacin. The susceptibility rates to these antibiotics before and after neomycin treatment did not significantly change. However, the MIC_50_ and MIC_90_ of neomycin against CRE increased after treatment, which could be attributed to several factors. At days 7 ± 2 and 14 ± 2, persistent CRE isolates with higher MICs were likely resistant to neomycin and unaffected by the treatment levels in the intestine. The possibility that neomycin exposure induced higher MICs in these persistent isolates cannot be ruled out. Interestingly, the susceptibility rates for gentamicin and amikacin remained consistent over time, suggesting a distinct resistance mechanism for neomycin [29].

Even though the side-effects of neomycin in this study were frequent, they were generally mild and self-limiting and did not necessitate specific intervention. Most participants with side-effects continued in the study and completed the neomycin course. Thus, future studies employing higher doses and longer neomycin treatments should ensure rigorous participant monitoring.

The strengths of this study include the following: (1) it is a randomized controlled study, (2) a neomycin-only regimen is used, and (3) neomycin exposure is not associated with the emergence of resistance to neomycin, gentamicin, and amikacin in CRE isolates.

The limitations of this study include the following: (1) this study is an open-label study without placebo in the control group, but a double-blind fashion with placebo in the control group is not necessary for assessing efficacy of neomycin in terms of the objective outcome, i.e., presence or absence of CRE from stool or rectal swab samples by laboratory methods, (2) there are some exclusion criteria including patients with severe renal impairment, patients receiving particular medications, and patients with GI diseases, resulting in a limited generalizability of the study results to these patients, and (3) this study does not assess clinically important outcomes, such as the rate of CRE infection in the participants who receive neomycin and the spread of CRE to other patients, health personnel and environment.

## 4. Materials and Methods

### 4.1. Study Design and Setting

This open-label randomized controlled trial took place from November 2022 to December 2023 at Siriraj Hospital, Bangkok, Thailand. The research was approved by the Institutional Review Board of the Faculty of Medicine Siriraj Hospital (reference: 660/2022) and registered at ClinicalTrials.gov (NCT05593601). The study adhered to the Declaration of Helsinki, and informed consent was obtained from all participants.

### 4.2. Participants

Eligible participants were hospitalized adults aged ≥ 18 years with a high risk of CRE colonization, as evidenced by the presence of CRE in stool or rectal swab samples. The high-risk factors included a history of CRE infection or colonization, hospitalization within the last 3 months, transfer from long-term care, recent broad-spectrum antibiotic use for Gram-negative bacteria, significant contact with CRE patients, care by a physician managing CRE cases, or hospitalization exceeding 2 weeks. The exclusion criteria were active CRE infection, the use of certain medications (cidofovir, colistimethate sodium, foscarnet, furosemide, digoxin), an estimated glomerular filtration rate less than 30 mL/min/1.73 m^2^, neomycin or aminoglycoside allergy, GI tract diseases, and pregnancy or lactation.

### 4.3. Detection of CRE

Stool or rectal swab samples from patients were processed in the infectious disease laboratory at Siriraj Hospital. The samples were cultured on mSuperCARBA agar plates (CHROMagar Ltd., Paris, France) and incubated at 35 °C for 18 h. Pink colonies indicated carbapenem-resistant *Escherichia coli* (*E. coli*), while blue or purple colonies signaled carbapenem-resistant (CR) non-*E. coli*, which were further identified for their species and tested for carbapenem resistance. The CRE isolates were stored at −70 °C for subsequent analyses. Carbapenemase types (OXA-48, NDM, and KPC) were identified via polymerase chain reaction. Susceptibility testing for neomycin was conducted via broth microdilution, adhering to an MIC threshold of ≤8 mg/L per the French Society of Microbiology guidelines. For gentamicin and amikacin, disk diffusion tests were performed, with interpretation based on zone diameters recommended by the Clinical and Laboratory Standards Institute [31].

### 4.4. Intervention

Participants were randomly assigned to either the neomycin group or the control group using stratified randomization by CRE species (*E. coli* or non-*E. coli*) at a 1:1 ratio in blocks of four. Those in the neomycin group received oral neomycin at 4.2 g/day (four 350 mg tablets, three times a day) for 5 days, while participants in the control group received no neomycin. The neomycin tablets (350 mg each) were provided by Greater Pharma Co Ltd., Bangkok, Thailand, which was not involved in the study’s design, data collection, analysis, publication decisions, or manuscript preparation.

### 4.5. Follow-Up of Participants

All participants hospitalized for ≥14 days were checked daily for adverse events, and those in the neomycin group were also checked daily for medication compliance. Stool or rectal swab samples were collected for CRE detection on days 7 ± 2 and 14 ± 2. Participants in the neomycin group discharged before completing the 5-day neomycin course took the remaining medication home, continuing for 5 days and reporting adherence and adverse events by phone until day 14. Those discharged before day 14 were provided with Cary–Blair transport tubes and verbal and written instructions for home stool sample collection on days 7 ± 2 and/or 14 ± 2. The tubes were mailed back for CRE testing.

### 4.6. Study Outcomes

The primary outcomes measured were the persistence rates of CRE in stool or rectal swab samples from the neomycin and control groups on days 7 ± 2 and 14 ± 2 post-randomization, alongside the safety and tolerability of neomycin. The study’s blind design ensured that the investigators remained unaware of the CRE detection results during the study. Secondary outcomes focused on the types of carbapenem resistance mechanisms (OXA-48, NDM, and KPC) in CRE isolates and their susceptibility to neomycin, gentamicin, and amikacin, analyzed using MedCalc Statistical Software version 19.6.4 (MedCalc Software Ltd., Ostend, Belgium; Available online: https://medcalc.org; 2021).

### 4.7. Sample Size Estimation and Statistical Analysis

The sample size was determined from a prior study indicating that 85% of hospitalized CRE-colonized patients remained colonized at discharge [11], with 66% of CRE isolates neomycin-susceptible [26]. Assuming that neomycin reduces colonization rates from 85% to 40%, calculations made with nQuery Advisor version 6.01 indicated that at least 22 participants were needed per group, considering a 5% type I error and 20% type II error.

Data analysis was conducted on a modified intention-to-treat (mITT) basis, including those with at least two sample collections and any neomycin dose administered in the neomycin group. Categorical variables were analyzed using the chi-square test or Fisher’s exact test, while continuous variables were compared via Student’s *t* test or the Mann–Whitney U test. A *p*-value < 0.05 was considered significant. IBM SPSS Statistics, version 20 (IBM Corp, Armonk, NY, USA) was used for the statistical analyses.

## 5. Conclusions

A 5-day course of oral neomycin at 4.2 g/day effectively reduced CRE colonization by day 7 ± 2. However, this effect waned by day 14 ± 2, indicating that the neomycin dosage and treatment duration may have been insufficient.

## Figures and Tables

**Table 1:** Baseline demographics and clinical characteristics of the modified intention-to-treat cohort.

Characteristics	Neomycin Group (*n* = 26)	Control Group (*n* = 26)	*p*
Male	8 (30.8%)	15 (57.7%)	0.05
Mean age (SD), years	63.6 (20.9)	71.7 (14.1)	0.11
Alive at discharge from hospital	22 (84.6%)	17 (65.4%)	0.11
Status before/on admission			
Underlying disease	22 (84.6%)	26 (100.0%)	0.11
Heart disease	4 (15.4%)	10 (38.5%)	0.06
Pulmonary disease	2 (7.7%)	7 (26.9%)	0.14
Hypertension	15 (57.7%)	18 (69.2%)	0.39
Diabetes mellitus	9 (34.6%)	10 (38.5%)	0.77
Renal disease	3 (11.5%)	7 (26.9%)	0.16
Cerebrovascular disease	11 (42.3%)	6 (23.1%)	0.14
Gastrointestinal and liver disease	2 (7.7%)	2 (7.7%)	1.00
Hematologic malignancy	2 (7.7%)	1 (3.8%)	1.00
Solid malignancy	2 (7.7%)	4 (15.4%)	0.67
Autoimmune disease	3 (11.5%)	1 (3.8%)	0.61
Median Karnofsky performance status scale (IQR)	50.0 (40.0–80.0)	50.0 (40.0–60.0)	0.32
Transfer from long-term care facility	2 (7.7%)	4 (15.4%)	0.67
Previous hospitalization within 3 months	15 (57.7%)	13 (50.0%)	0.58
Urinary catheter	1 (3.8%)	4 (15.4%)	0.35
Nasogastric tube	3 (11.5%)	6 (23.1%)	0.47
Tracheostomy tube	5 (19.2%)	5 (19.2%)	1.00
Previous major surgery within 3 months	2 (7.7%)	1 (3.8%)	0.55
Previous antibiotic use within 3 months	15 (57.7%)	15 (57.7%)	1.00
Previous immunosuppressive agent use within 3 months	4 (15.4%)	4 (15.4%)	1.00
Previous CRE infection	0 (0.0%)	1 (3.8%)	1.00
Previous CRE colonization	2 (7.7%)	4 (15.4%)	0.67
Status during admission			
Indwelling central intravascular line	5 (19.2%)	3 (11.5%)	0.70
Mechanical ventilator	13 (50.0%)	14 (53.8%)	0.78
Indwelling urinary catheter	21 (80.0%)	21 (80.0%)	1.00
Major surgery	5 (19.2%)	0 (0.0%)	0.05
Chemotherapy	3 (11.5%)	0 (0.0%)	0.24
Diagnosis			
Heart disease	2 (7.7%)	4 (15.4%)	0.67
Pulmonary disease	2 (7.7%)	6 (23.1%)	0.25
Renal disease	2 (7.7%)	3 (11.5%)	1.00
Cerebrovascular disease	5 (19.2%)	1 (3.8%)	0.19
Hematologic malignancy	2 (7.7%)	1 (3.8%)	1.00
Solid malignancy	4 (15.4%)	1 (3.8%)	0.35
Autoimmune disease	3 (11.5%)	0 (0.0%)	0.24
Previous or current documented or presumed infection during this admission up to the end of the study	21 (80.8%)	26 (100.0%)	0.05
Had received or receiving antibiotics during this admission up to the end of the study	26 (100.0%)	26 (100.0%)	1.00
Species of CRE isolates	28 CRE isolates	28 CRE isolates	
*E. coli*	8 (28.6%)	10 (35.7%)	0.78
Non-*E. coli*	20 (71.4%)	18 (61.5%)	0.78
Susceptibility of neomycin against 28 CRE isolates	26 (92.9%)	26 (92.9%)	1.00
Susceptibility of gentamicin against 28 CRE isolates	14 (50.0%)	13 (46.4%)	0.80
Susceptibility of amikacin against 28 CRE isolates	13 (46.4%)	15 (53.6%)	0.80
MIC_50_ of neomycin against 28 CRE isolates	2 mg/L	2 mg/L	
MIC_90_ of neomycin against 28 CRE isolates	8 mg/L	8 mg/L	
MIC range of neomycin against 28 CRE isolates	1–32 mg/L	1– > 128 mg/L	

**Table 2:** Neomycin decolonization efficacy against carbapenem-resistant *Enterobacterales* (CRE) in the modified intention-to-treat cohort.

Persistence of CRE Fecal Carriage	Neomycin Group (*n* = 26)	Control Group (*n* = 26)	*p*
Day 1	26 (100.0%)	26 (100.0%)	
Day 7 ± 2	12 (46.2%)	21 (80.8%)	0.01
Day 14 ± 2	19 (73.1%)	16 (61.5%)	0.56
		21 (80.8%)	0.74

**Table 3:** Susceptibility of carbapenem-resistant *Enterobacterales* (CRE) to neomycin, gentamicin, and amikacin across study timepoints in both study groups.

Susceptibility of CRE to	Neomycin Group	Control Group	*p*
neomycin on day 1	26/28 (92.9%)	26/28 (92.9%)	1.00
neomycin on day 7 ± 2	12/14 (85.7%)	20/22 (90.9%)	0.64
neomycin on day 14 ± 2	18/22 (81.8%)	15/17 (88.2%)	0.68
*p*	0.50	0.87	
gentamicin on day 1	14/28 (50.0%)	13/28 (46.4%)	1.00
gentamicin on day 7 ± 2	7/14 (50.0%)	10/22 (45.4%)	0.94
gentamicin on day 14 ± 2	12/22 (54.4%)	9/17 (52.9%)	0.82
*p*	0.94	0.95	
amikacin on day 1	13/28 (46.4%)	15/28 (53.6%)	0.79
amikacin on day 7 ± 2	6/14 (42.9%)	9/22 (40.9%)	0.82
amikacin on day 14 ± 2	12/22 (54.5%)	9/17 (52.9%)	0.82
*p*	0.76	0.63	

## Data Availability

The study dataset used in this study is available from the corresponding author upon reasonable request.
